# Digital personal assistants are smart ways for assistive technology to aid the health and wellbeing of patients and carers

**DOI:** 10.1186/s12877-021-02436-y

**Published:** 2021-11-15

**Authors:** Gayathri Victoria Balasubramanian, Paul Beaney, Ruth Chambers

**Affiliations:** 1 Centre for Biomechanics and Rehabilitation Technologies, Faculty of Health, Staffordshire University, Stoke-on-Trent, UK; 2 Keele University Medical School, Keele, Newcastle-under-Lyme, UK; 3 Digital Workstream, Staffordshire and Stoke-on-Trent Sustainability and Transformation Partnership (STP), Staffordshire, UK

**Keywords:** Assistive device, Assistive technology, Health, Healthcare, Diabetes, Long-term condition

## Abstract

**Background:**

Digital health solutions such as assistive technologies create significant opportunities to optimise the effectiveness of both health and social care delivery. Assistive technologies include ‘low-tech’ items, such as memory aids and digital calendars or ‘high-tech’ items, like health tracking devices and wearables. Depending on the type of assistive devices, they can be used to improve quality of life, effect lifestyle improvements and increase levels of independence. Acceptance of technology among patients and carers depends on various factors such as perceived skills and competencies in using the device, expectations, trust and reliability. This service evaluation explored the impact of a pilot service redesign focused on improving health and wellbeing by the use of a voice-activated device ‘smart speaker’, Alexa Echo Show 8.

**Methods:**

A service evaluation/market research was conducted for a pilot service redesign programme. Data were collected via a survey in person or telephone and from two focus groups of patients (*n* = 44) and informal carers (*n* = 7). The age of the study participants ranged from 50 to 90 years. Also, the participants belonged to two types of cohort: one specifically focused on diabetes and the other on a range of long-term health conditions such as multiple sclerosis, dementia, depression and others.

**Results:**

The device had a positive impact on the health and social well-being of the users; many direct and indirect benefits were identified. Both patients and carers had positive attitudes towards using the device. Self-reported benefits included: reminders for medications and appointments improved adherence and disease control; increased independence and productivity; and for those living alone, the device helped combat their loneliness and low mood.

**Conclusion:**

The findings from the study help to realise the potential of assistive technology for empowering supporting health/social care. Especially, the season of COVID-19 pandemic has highlighted the need for remote management of health, the use of assistive technology could have a pivotal role to play with the sustainability of health/social care provision by promoting shared care between the care provider and service user. Further evaluation can explore the key drivers and barriers for implementing assistive technologies, especially in people who are ageing and with long-term health conditions.

## Background

The growing burden of chronic diseases is one of the most frequently stated public health problems. With an ageing population in the UK, the burden of chronic medical conditions is expected to increase. For instance, the number of people with diabetes in the UK currently are 4.9 million people and it is expected to increase to 5.5 million people by 2030 [1, 2]. Another persisting public health concern is dementia which affects an estimated 850,000 people in the UK [3]. Many people with dementia require carers and much of the care for persons living at home is provided by informal carers [4]. In the UK, there are over 700,000 informal carers for persons with dementia [4, 5]. Not only do these diseases impose a huge health burden, but also a socioeconomic burden. National Health Service (NHS) spending has substantially increased for the management of chronic health conditions; the total expenditure in 2019 on long-term care (health and social) was increased by 5% in nominal terms in comparison to what was in 2018 in nominal terms [6]. This only highlights a small portion of the total health burden. Long-term health conditions can significantly reduce the quality of life. In addition, as mental and physical health conditions are closely linked, health care needs are more complex. In order to meet complex health care requirements, there is a growing body of literature that focuses on the need for innovation and the use of technologies to assist those living with them [7–11]. Digital health solutions, especially in the form of assistive technologies create significant opportunities to optimise both health and social care delivery. Many studies support the idea that digital health technologies can transform and complement conventional methods of health care provision, and thereby reduce demand on local services [12–14].

Acceptance of technology among patients and carers depends on various factors such as perceived skills and competencies in using the device, expectations and reliability [15, 16]. Research shows that the perceived level of effort required to use the device and overall utility of the device affects its adoption and use [16]. Besides ease of use, availability of support measures such as organisational infrastructure, customised interventions to support tool adoption and availability of individualised IT support had a positive effect on the perception of usefulness and on understanding a technology enabling individuals to adjust to new techniques quickly [16]. This shows that adoption and use are subjective and varies depending on the device and its applications.

Assistive technology is any device or system that allows an individual to perform a task that they would otherwise be unable to do or increases the ease and safety with which the task can be performed. Assistive technology includes a wide range of devices from simple ‘low-tech’ items, such as memory aids and digital calendars to more ‘high-tech’ items, like health-tracking devices and wearables. Depending on the type of assistive devices, they can have a wide range of applications and be used as an effective device to improve quality of life, effect lifestyle changes and increase the level of independence [17–21].

This pilot study aimed to explore the user experience of one such device. This device is a compact tablet with a screen and speaker with voice-control that relays personal digital assistance with various built-in skills that have a wide range of applications. This study intended to understand the assistive device’s potential to support ordinary people’s everyday living and potential impact on their health and wellbeing in real-world settings.

## Methods

In order to explore the impact of assistive-technology a service evaluation was conducted for a pilot service redesign programme. The patients and informal carers were recruited using a non-probability, convenience sampling technique through select locations: Burton diabetes patient network, Home Instead Senior Care and a GP practice in Northern Staffordshire. The target population invited and recruited through convenience sampling was based on the location, internet service and the network of the researchers. Participants were invited through either email or word of mouth. They were referred into the programme by multiple pathways such as diabetic support groups, GPs, social workers, care agencies and improving access to psychological therapy (IAPT) services. As a part of the programme, a voice-activated assistive device with a screen and built-in Alexa skills was provided to patients (the Alexa Echo Show 8). Participants had to fulfil certain criteria to use the device for a health benefit for at least 2 months and provide feedback. They had to have their own means of internet supply and be willing to set up the device by themselves or with the help of a friend or a family member. Many of the recipients had never used a smart speaker before let alone considered buying one. Some were more tech-savvy than others, but most were new to this type of technology (voice-activated assistive technology). The devices were installed during the period February 2019 to December 2019 at the participants’ homes. Initially, support for installation and demonstration of the device was provided. Once the patients started using the device for some time, they were invited to participate in a telephone survey and/or a focus group interview. Some of the participants of the telephone survey also were a part of the focus group. Especially, participants who were recruited through Home Instead Senior Care, after the installation of the assistive device in their homes, the participants were surveyed over the phone. The telephone survey and the two focus groups were conducted to gain new insights into the acceptance and user experience to gauge the use of assistive technology for health and social wellbeing. Key questions from the interview guide and survey are presented in Table 1 below.

**Table 1:** Key questions from survey and interview guide


**Key questions asked to patients:**	
• In terms of difficulty, how would you rate your experience of the assistive device Alexa?	
• What made it ‘difficult’, ‘so-so’ or ‘easy’ for you?	
• What words come to mind when thinking about the effect of Alexa on your health and well-being?	
• How would you best describe your social activity before and after the project?	
• How often did you use Alexa?	
• As an aid to improve your health & wellbeing, what is good about Alexa?	
• What could be better?	
• Please rate how using Alexa affected your independence? How?	
• What surprised you about using Alexa?	

**Key questions asked to carers:**	
• What words come to mind when thinking about the effect of Alexa on [insert name here]‘s health and well-being?	
• How has Alexa helped you to provide support to [insert name here]?	
• How often did you use Alexa?	
• What effect has using Alexa for [insert name here]‘s health & wellbeing had on them? On you?	
• What are the main advantages of using Alexa compared to the way you previously assisted with healthcare? What are the disadvantages?	
• As a health tool, what is good about Alexa? What could be better?	
• What surprised you about using Alexa?	

Icons made by Freepik [22]. Available at: https://www.flaticon.com (Accessed: 11 July 2020)

The survey response rate was 88%. The two focus groups conducted were exclusively for patients with diabetes (both types 1 and 2), but most had existing comorbidities. Focus groups conducted in Burton and Tunstall included 23 and 4 attendees, respectively. The opinions collected through telephone surveys and the focus groups from both patients (*n* = 44) and their informal carers (*n* = 7) same as patient responders or on behalf of other patient recipients were collated and analysed. The age of the participants ranged from 50 to 90 years. The patients who participated in the survey had various medical conditions such as diabetes, dementia, Parkinson’s disease, asthma, Behçet’s disease, Cushing’s syndrome, phenylketonuria, liver disorders, low mood, depression, anxiety, dyslexia, cognitive impairment, severe visual impairment with a disability to read and write, chronic knee pain affecting mobility and trauma. A flexible approach was taken towards exploring a wide range of data gathered through the phone survey and focus groups using a mixture of open and closed questions (Table 1). As the goal for collecting qualitative data (mainly from the open-ended questions and field research notes) was to gain an understanding of particular outcomes from the perspective of those experiencing it., content analysis was performed to identify general themes, synthesise information and interpret the data to find outcomes and associations. Since the data set was small statistical packages such as NVivo or QDA data minor were not used. The data was initially extracted on to EXCEL forms and cleaned thoroughly. As a next step for content analysis, the data was grouped based on the presence of certain words, themes, or concepts within the text. This data was scrutinised and analysed by the researcher to find meanings and relationships of certain words, themes, or concepts. Furthermore, if a pattern or a relationship was identified, the data was evaluated to be observed for consistency before making inferences about the messages within the texts. The results were tabulated in EXCEL and then transferred to a WORD document, where the data was organised into paragraphs of information based on the themes such as self-management and autonomy, impact on the lifestyle habits and impact on the mental and social well-being of the patients. Quotes from the patients and carers were also captured on a table for direct reference.

## Results

**Fig. 1:**
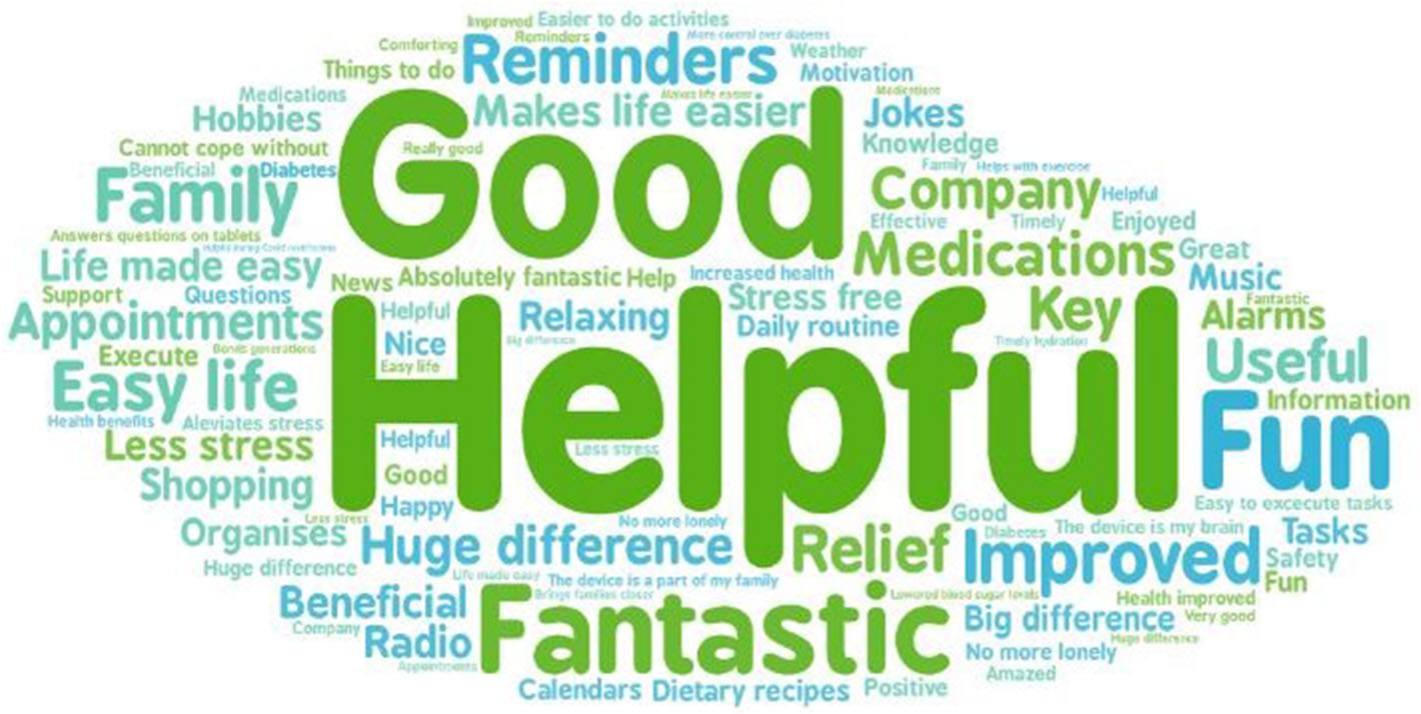
Word cloud with descriptive words showing user experiences

Overall, both the patients and the carers had a positive experience with the assistive device. The word cloud below (Fig. 1) summarises the words used to describe the user experience by the patients and carers.

The results from the diabetes focus groups showed that around 34% of the patients used the device for diabetes-related support, and 32% for general support. The remainder of the patients had various types of medical conditions. Around 91% (40 out of 44) of the patients used the device daily. The use of the device was measured based on users’ self-reported outcomes. The participants had the device installed in their home for at least 2 months before they received the evaluation phone call. Self-reported outcomes based on at least 2 months use of the device were preferred as it may be difficult to gather this data in a way that would be acceptable to most people without raising privacy concerns or ethical issues. The vast majority of users found the device was easy to use. Many (57%) found that voice control made the device easier to use (Quote 1) and the self-perceived technological competency was associated with the rating of experience. On the other hand, speech difficulties interfering with voice commands and at times, delayed voice activation made the experience difficult for some users. Through repetitive learning/practice over time and with some help, most of the users have integrated the device into their lifestyle. In general, the patients and the carers had an optimistic attitude towards the use of the assistive device and found that it had a positive impact on their health and well-being (Fig. 1).

Firstly, the device was found to be useful for organising the lives of the patients. Due to various medical conditions, many patients had challenges (some more severe than others) with remembering appointments, cooking and even performing daily activities such as emptying bins (Table 2: Quotes 1 to 4). Moreover, forgetting to switch off cookers or ovens poses serious health and safety issues. But, as shown in quote 3 the assistive device helped overcome such challenges through timely reminders. With the assistive device, the users were more independent, and their living situation improved considerably. The device helped with planning and organising and setting reminders for appointments with General Practitioners/hospitals or laboratories, medications intake, injections and food or drink consumption, which had an overall positive impact on the lives of patients. Timely consumption of medications and adherence to treatment is essential for those with chronic conditions. Thus, as shown in quotes 3 and 4 the assistive device had an indirect effect on the health of the patients by reminding the time for medications and appointments. Furthermore, the device alleviated stress and anxiety related to missing medications and appointments. From a carers’ perspective, many felt a sense of reduced pressure, reassurance and peace of mind as the device aided the patients to keep up with appointments or activities and reduced the need for frequent visits or checking from carers. The carers also perceived that the patients had increased independence and decreased anxiety with the help of the device. Most carers felt that with the device, they had the reassurance of safety and welfare of the individuals they were caring for (Table 2 Quote 5).

**Table 2:** Quotes from patients and carers on the use of assistive device

	Example responses from patients and carers
Quote 1	“Getting information is good (if it is able to) – medical, general knowledge. If you’re not computer literate it makes info much more accessible! Used it for reminders to take medications, remind day before appointments.”
Quote 2	“Alexa has helped her to get to her appointments that she was always missing before – warns before she has to go, sends dual reminders to mum’s phone. Helps her to remember to prepare for her son’s activities e.g. make her son’s sandwiches. Helped son with education. Son has gotten very involved. Tells her to lock the door. Helped with cooking. Increased independence, reduced anxiety, peace of mind, learning new skills.”
Quote 3	“Memory is not very good so was always missing appointments, unable to cook even simple meals as would forget to switch oven on/off - now she can with her son and Alexa’s help – reminds her to take things out of the oven and switch it off. Running life more smoothly.”
Quote 4	“Good to ask medical questions – reminder to take tablets is useful”
Quote 5	“Positive for both carers and patient. Independence for carers too.”
Quote 6	“Small and neat. Not used it for diabetes per se. Ask questions about current affairs, play music, wants to use it for meditation, had a gastric band fitted. Would like to be able to keep a food diary with a calorie count. Set exercise targets. Finds it friendly and comforting – can ask it questions, say good night etc. Husband uses it for knowing where she is (in a good way!) – it is linked to her diary.”
Quote 7	“When taking pain medication she can’t sometimes remember if she has taken it or not – Alexa relieves this anxiety for her as she tells it when she takes any medication so she can get it to remind her later; getting recipes for her intolerances easily.”
Quote 8	“Type 1 DM - Carb content of food – easier than googling, counts carbs and converts it into insulin units (separately) – not perfect, sometimes uses US units i.e. cups etc. Uses it as a calculator function to work out insulin ratio from it. Dyslexia – spelling words for wife.”
Quotes 9	a) “Thinks it has improved health: more motivated because don’t forget things as much, alarm clock gets him out of bed, feels less frustrated with himself. Been great to have at the moment because of Covid-19!” b) “Found it fun, really helpful for covid-19 restrictions, very comforting to have in the house”. c) “Using it for music, quizzes. Used it to keep in touch with granddaughter. Exercises have been really good to do some exercise especially with Covid-19”.
Quote 10	“Keeping track of diary, putting bins out, meditation, helps with stress, helps with loneliness – keeps her company; play music.”
Quote 11	“How easy it is – thought it would be difficult to set up – could have done it herself it was so easy; surprised at the amount she uses it – and it’s good for company too – asks for a fact or a joke randomly - although her jokes are bad! Surprised at just how positive it has been for her.”
Quote 12	“Gives mum a lot of reassurance and peace of mind. Thinks it has changed Mr. X’s daily life for the better.”
Quote 13	“Gone from 24 h care in XYZ House to live by himself in his house; medication reminders in particular meant that he could keep his mental health problems under control and could be safe to himself and others; set it up by himself too. Music for relaxation.”

Icons made by Becris (2020). Available at: https://www.flaticon.com (Accessed: 11 July 2020); Quotes 9: a, b and c were from three different participants in the study

Secondly, the device had a positive impact on the lifestyle habits (mainly diet and exercise) of the users. As seen in quotes 6, 7 and 8 (Table 2), patients benefitted by obtaining recipes tailored to their personal requirements based on dietary intolerances, health conditions (e.g. diabetes), surgical history (e.g. with gastric banding), weight management issues and such. Lifestyle choices and weight management is a major issue, especially in people with diabetes. But, with the assistive device patients benefitted from easily obtaining diabetes-friendly recipes for cooking and baking. The users were able to enquire about the calorie content and carbohydrate content in various foods (Table 2 Quote 8). Furthermore, through the interactions with the device, patients were able to access information on swapping high calorie/sugar-rich ingredients with ones with less calorie/sugar in no time. Besides diet, users were able to watch videos and perform exercises, set targets for effective weight management, and engage in meditation and stress-relieving activities (Table 2 Quote 6). All of these mentioned activities are known to improve the health of people with diabetes. People with diabetes felt that they had better control over their blood sugar levels. A few mentioned that their blood sugar levels were reduced since the use of the device and were able to switch from taking fast-acting insulin. This may be attributed to better adherence to treatment, health education and improved self-management of diabetes. Not only in people with diabetes but also other users found the device to be helpful for healthy living through useful tips on diets and exercise. The assistance with exercise has been advantageous particularly during the lockdown situation owing to the Coronavirus (COVID-19) outbreak (Table 2 Quotes 9 a, b and c). Overall, the device had aided to positive behavioural change, which in turn may have indirect benefits to physical and mental well-being in the long run.

Thirdly, the device had a positive impact on the mental and social well-being of the patients. Many patients with chronic comorbidities suffer from emotional health issues, which can be detrimental and further worsen their overall health and well-being. For instance, recently diabetes and emotional health has been a widely discussed topic. Patients with diabetes have various types of psychological problems such as diabetes distress, fear of hypoglycaemia/insulin, depression, anxiety disorders, frustrations due to sexual dysfunction and eating disorders [23, 24]. For such patients, digital personal assistant like Alexa can be useful to overcome some of the psychological or emotional health challenges. In this study, users found that engaging in conversation with the voice-activated device helped to combat loneliness, low mood and depression (Table 2 Quotes 10 and 11). Engaging in simple conversations about the weather, news, general knowledge and latest information made a huge difference to the users. Moreover, patients could ask the device medical questions and side-effects of drugs and feel better informed. Users with visual impairment benefitted from the voice-based features the most. As shown in quotes 11 to 13 (Table 2), simple features such as radio, music players, video players, shopping, games, quizzes and telling jokes provided a sense of companionship to the users.

A few users were able to pursue their hobbies and even learn new skills through the device’s assistance. It was mentioned that the device helped with sleep by playing music or sounds of nature or ocean waves. Likewise, some users used the device to take virtual holidays and watch relaxing videos of nature, which had a calming and soothing effect on them. This demonstrated the potential of the device to improve mental well-being. Patients also connected with carers and family members through free audio and video calls. Furthermore, users felt that technology brought the family members closer and even facilitated bonding across generations. Many of the users found that their day-to-day living experience has improved with the use of the device for various reasons. Many found that their social activities increased, and mental health has improved/managed effectively since using assistive technology (Table 2 Quotes 10 and 13). Besides, the views of the carers’ corroborated with those of the patients as they could see the positive impact of the device on the social and mental well-being of the users, especially in people who were ageing or had mental health issues.

## Discussion

This study explored the everyday use of technology by patients with various medical conditions and their family carers, and how they integrated the technology into their daily routines, including key factors which influenced this. The vast majority of the patients, as well as their carers, expressed a positive experience of the assistive device. In line with many other research findings in the area, some of the common reasons for using the device were: ease of use, assistance with planning various tasks and accessing information [4, 25–27].. Additionally, the device provided a sense of reassurance, companionship, comfort, security and autonomy [4, 25–27].

Some of the results defied the common perception that older individuals are apprehensive about technology [15]. Previous research in this area has shown that the ageing population feared technology and were less likely to use them [15, 28]. Generally, the acceptance of new digital solutions and innovative technology by patients and carers relies on their anxieties and feelings of insecurity concerning the device/technology. Our findings suggest that most of the users (geriatric population functioning in different levels of independence) recruited through senior care providers, a diabetes network and GPs were eager to adopt new technology and willing to learn to use them, which accords with previous research in a similar area [29]. However, some patients did voice concerns in terms of difficulty in using the device. This was mainly due to their self-perceived technological incompetency and initial feelings that they were not “tech-savvy”. Consistent with the literature [26, 29], although the patients found the device complex and voiced apprehension about a lack of clarity, instructions and support initially, over time they learnt to use it. Eventually, upon achieving successful integration of the assistive device into their daily lives, they harnessed its potential. This is reflected in their responses, which shows that the device made their life a bit easier. Additionally, the carers expressed that they were surprised about the use of the device for providing support by answering key questions that they had, helping them with reminders, and organising their daily life as it is not advertised directly for this purpose. The carers recognised the fact and suggested that it was important to tap the potential. These findings show that assistive technology can enable people to have more control over their lives, health, social and mental well-being. Furthermore, it can assist the carers with their roles and responsibilities.

The present study raises the possibility that assistive technology besides offering general support, may benefit patients with chronic medical conditions such as diabetes and dementia. For instance, effective diabetes care is largely dependent on patient self-management and patient empowerment. Patients with diabetes may have to seek knowledge and need to sift through many sources to find information to make essential lifestyle choices concerning diet and exercise [7]. Moreover, they need assistance with monitoring blood glucose levels, strictly adhering to medications, and determine their insulin dose and timing [30]. The findings generated from this study showed that the assistive device played an effective role in providing this support. Patients were self-motivated and driven towards their goals to manage their health with that extra personal support. As suggested in previous research, this shows that digital health and assistive technologies may help improve patient outcomes [30, 31]. On the other hand, certain medical conditions like dementia require a lot of carer support. Carers feel the need to be informed with the latest knowledge through a variety of sources to find out about the support available for the person with the medical condition and to enhance their care levels. They also have constant concerns about the safety and well-being of those whom they care for. The findings from this study showed that the assistive device supported the carers in this regard. These results seem to be consistent with existing research, which found that assistive technology can provide reassurance and support for carers of persons with dementia [4].

Whilst the use of assistive technology is being well-realised, there are a few barriers to adoption in the mainstream of health care. First of all, individuals accept change at different rates and the process of adapting to technology in their lives takes time. As discussed earlier, self-perceived technical skills and also the real ability to learn and develop skills to use the device remains a key challenge. This can be overcome by understanding the users’ perceptions of technology while introducing digital solutions to this population. This will lead to the preparation of an extensive user community to successfully implement digital solutions at a community level and maximise the potential of technology to facilitate independent living. Internet availability, especially wi-fi speed may be an additional factor that may be a challenge to access and use assistive technologies unhindered. Currently, there is increased concern about ethical issues whilst adopting health technologies [32]. Alongside healthcare provision, protection of privacy of patient data has always been a priority with healthcare providers and technology developers. Through careful consideration and well-devised regulatory safeguards and boundaries patients’ data can be protected, which in turn would facilitate the adoption of technology for various beneficial purposes [32]. Also, educating users on data protection and providing useful instructions to protect their privacy can have mutual benefits for both providers and users. Another most common barrier to adoption is cost [32]. The costs of the device and maintenance can be an issue with implementation for both the individuals and organisations. It may be true that investing in good assistive technologies can incur substantial costs, especially for health care providers. However, the potential benefits may outweigh the onetime set-up and maintenance costs. The costs of adoption to assistive technology for improving health and social well-being may be lesser in comparison to paying the price owing to increasing health and socioeconomic burden leading to unmet needs. Constantly, there is an increasing demand on local services and a significant burden is imposed on health care workers. This results in unmet needs due to the national shortage in health care workers and carers [26, 33–35]. However, such situations can be better managed by embracing assistive technologies as an adjunct for care provision and remote monitoring that can ease some of the workloads of health care workers/carers [7]. This, in turn, may have a positive impact on their health and well-being as well as improve productivity. Thus, there may be some direct and indirect cost-effectiveness with the adoption of assistive technology. There is some evidence in the existing literature that institutional and certain in-home personnel costs reduced through strategic implementation of assistive technology for care [36, 37]. However, continued research in the area to assess the cost-effectiveness of assistive technologies for health and social care is required specific to devices and settings.

Another widely debated concern is whether technology promotes or demotes social interactions. Current literature highlights both positive and negative impact of technology on social interactions. Whilst few studies highlight that new technologies can have a dehumanizing effect of distance and create insensitivity, some suggest that it facilitates social interactions [38–40]. In general, evidence suggests that technology may have negative impact on children and young adults [38, 41]. However, for adults who enter into older adulthood, use of social media through various technologies may play a more active role in keeping this geriatric population socially connected [42]. This is because maintaining social connectedness may become cumbersome or increasingly difficult due to mobility limitations, chronic diseases and other age-related issues, thus decreasing physical connectedness with friends, family, and community [40, 42]. Similarly, many of our participants found that their digital assistants facilitated social contact (for the socially isolated, patient’s carers, distant family and friends) rather than substituting it. Since the device also had a screen, making video calls was easier through voice-commands which were found to be helpful for many. As highlighted earlier, the assistive device facilitated a bonding experience across generations for some of our participants. Moreover, in the recent times, during various national lockdown restrictions in the UK owing to the COVID-19 pandemic, the assistive device was found to be timely. Exploring the benefits of technology for social interactions, especially in geriatric population may be an interesting avenue of future research projects.

### Strengths and limitations of this study

This study recruited participants (patients and carers) from a select region in the UK through a convenience sampling strategy to identify people with a range of medical conditions to capture data relevant to the use of a particular type of assistive device and to explore user experience post the integration of the device in their routine lives. The findings from the study provided rich insights from real-world data to understand patients’ and carers’ perspectives on using assistive technology. Many valuable insights were generated on how assistive devices integrated into the routine lives of individuals and played the role of a personal assistant. Furthermore, the study helped to identify some enablers and barriers to integration of assistive devices in routine care, which can support with health care decision-making on future implementation of assistive technologies in homes, effectively facilitated by formal health and social care services. A limitation of the study could be the small sample size limited to one geographical area of England, so our findings may have limited generalisability to other locations. The participants were recruited using convenience sampling method, which involves using respondents who are accessible to the researchers. Therefore, there may be some degree of selection bias. Future research in larger sample using probability sampling techniques may help to overcome such bias.

## Conclusion

Assistive technology has become one of the most powerful tools in supporting people in their home, which is gaining attention worldwide [12, 14]. This study explored the user experience of an assistive device for routine aid in promoting their health & wellbeing and self- care. The findings from the study showed that the device had a positive impact on the health and social well-being of the users; many direct and indirect benefits were identified. The acceptance of technology was good in both patients and carers as they had a positive attitude towards using the device.

Firstly, the findings from the patients’ responses showed that reminders for medications and hospital appointments meant self-reported improvements in adherence and compliance to treatment. Most patients felt they made them more independent and productive. Additionally, for those living alone, the device helped combat perceptions of loneliness and depression. Additionally, it also facilitated patients to take more responsibility for their health and wellbeing. Secondly, the carers also perceived that the device improved the physical and mental wellbeing of the patients. Overall, the findings from the study help to realise the potential of assistive technology for supporting health and social care by aiding treatment compliance/adherence, better self-management, improved mental health and patient autonomy. Further research and evaluation can explore the key drivers and barriers for implementing assistive technologies as an adjunct to existing care models for optimal management of health care needs, especially in people who are ageing and with certain chronic medical conditions. Not only the patients and carers, but assistive technologies can benefit the healthcare services as well. Constantly, there is an increasing demand on local services, which is worsened in times of crisis such as the current COVID-19 pandemic. In such a time as this, the service provided using the assistive device may be of help to ensure the sustainability of health and social care provision by promoting shared care.

## Data Availability

The datasets during and/or analysed during the service evaluation/market research study is available from the authors on reasonable request.

## Abbreviations

COVID-19: Coronavirus 2019
NHS: National Health Service
IT: Information technology
STP: Sustainability & Transformation Partnership
